# A start methionine mutation in matriptase causes the membrane anchor to be skipped and replaced by a cryptic signal peptide

**DOI:** 10.1016/j.jbc.2026.113496

**Published:** 2026-08-26

**Authors:** Monika Mrackova, Maiken Jaller Jensen, Annika Weile Nonboe, Signe Skovbjerg, René Villadsen, Lotte Katrine Vogel

**Affiliations:** Department of Cellular and Molecular Medicine, Faculty of Health and Medical Sciences, University of Copenhagen, Copenhagen N, Denmark

**Keywords:** serine protease, eukaryotic translation initiation, trafficking, cryptic signal peptide, start-loss mutation, ARCI11, matriptase, Ally protein

## Abstract

The serine protease matriptase is expressed on the plasma membrane of many cell types. Matriptase knockout mice die shortly after birth, whereas in humans, homozygosity for a start-loss mutation (matriptase M1I) is compatible with life. Because the remaining in-frame methionine codons are located downstream of the transmembrane domain, matriptase M1I is predicted to lack its membrane anchor and, at best, become nonfunctional due to mislocalization to the cytosol. This study aims to determine whether matriptase M1I is produced as a correctly located potentially functional protein. Our findings show that matriptase M1I can be expressed in a cell model as a truncated protein detectable by antibodies against matriptase. Using cell-surface biotinylation and streptavidin purification, we demonstrate that matriptase M1I is present on the plasma membrane and exhibits matriptase-specific enzymatic activity. Bioinformatic analyses predict the presence of a non-cleavable cryptic signal peptide downstream of the methionine residue at position 180 (wild-type matriptase numbering), resulting in the same membrane topology as wild-type matriptase. Initiation of translation from this alternative start codon is predicted to generate a membrane-bound protein lacking the cytosolic tail, the transmembrane domain, and the SEA domain of wild-type matriptase, while gaining a non-cleavable signal peptide derived from a sequence that normally forms part of the SEA domain in wild-type matriptase. Thus, it seems likely that the human genetic variant matriptase M1I is expressed as an enzymatic active protein with the correct subcellular localization and thus may substitute for the essential function of wild-type matriptase.

Matriptase is an essential protein in mice, as matriptase knock-out mice die shortly after birth (1). In contrast, homozygosity for a start-loss mutation (matriptase M1I) is compatible with life in humans (2). Matriptase M1I is predicted to lose its membrane anchor due to the location of the remaining in-frame methionine codons, suggesting that at best the protein becomes nonfunctional due to mislocalization to the cytosol. The present study aims to elucidate whether the M1I mutation produces a matriptase-like protein product and whether it possesses similar biochemical features to wild-type matriptase that could explain how human carriers of the M1I mutation can survive.

Matriptase M1I is one of a range of genetic alterations in the gene encoding the serine protease, matriptase, that is known to cause a syndromic form of ichthyosis called Autosomal Recessive Congenital Ichthyosis 11 (ARCI11) (2, 3, 4, 5, 6). ARCI11 is a mild condition and not connected to a reduced life expectancy. Elucidating the mechanism leading to the development of ARCI11 is beyond the scope of this study.

Matriptase is an 855 amino acid residue transmembrane, multi-domain serine protease containing, from the N-terminus: a short cytoplasmic tail, a transmembrane domain followed by a sea urchin sperm protein, enteropeptidase, and agrin (SEA) domain, two complement C1r/s urchin embryonic growth factor and bone morphogenetic protein-1 (CUB) domains, four low-density lipoprotein receptor (LDLR) domains, and a carboxy-terminal serine protease domain (SPD) (Fig. 1). During its synthesis, matriptase is co-translationally translocated across the ER membrane and transported down the secretory pathway to the plasma membrane.

**Figure 1:**
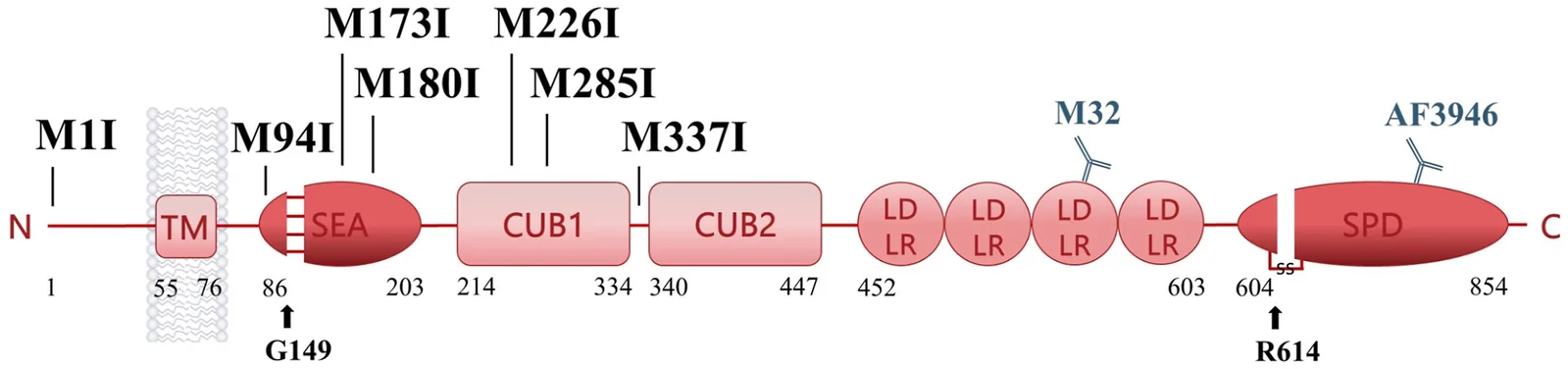
**Six possible alternative in-frame start codons were identified for matriptase M1I.** Schematic representation of the matriptase protein. Matriptase consists of a short cytoplasmic N-terminal segment, a transmembrane domain (TM), a SEA domain followed by two CUB domains, four LDLR domains, and an SPD. The ichthyosis-causing M1I variant as well as the six downstream methionine residues in the same reading frame (M94, M173, M180, M226, M285, and M337) are indicated. The amino acid numbering of each domain and the epitopes of the antibodies M32 and AF3946 are indicated. Matriptase is cleaved after G149 (SEA-domain cleavage) and after R614 (activation), as indicated by *arrows*.

Matriptase is highly conserved from fruit flies to primates. Using mouse models, it has been shown that uncontrolled matriptase activity is highly carcinogenic, since 70% of mice overexpressing matriptase under the K5 promoter develop malignant skin cancer (7). In contrast, mice overexpressing both matriptase and either of the Kunitz-type matriptase inhibitors, hepatocyte growth factor (HAI)-1 or HAI-2, have a cancer incidence close to that of wild-type mice (7, 8). Matriptase can be found on the basolateral plasma membrane of epithelial cells (9), where it conveys pro-oncogenic signals that are well known to play a crucial role in carcinogenesis, for example through the protease-activated receptor 2 (PAR2) (10) and HGF (11) pathways. Matriptase belongs to a small group of proteolytic enzymes in which the zymogen form exhibits significant proteolytic activity. We have recently shown that the zymogen activity can be inhibited by the same inhibitors that targes the activated form of matriptase, such as HAI-1 or HAI-2 (12).

HAI-1 and HAI-2 also function as ally proteins (13, 14, 15, 16). The ally function is not well understood; it describes the fact that full-length matriptase is not detectable on the cell surface or in cell lysates unless HAI-1 or HAI-2 is present/co-expressed. This phenomenon has previously been described as a chaperone-like function, but there are no indications that the absence of HAI-1 or HAI-2 causes proteasomal degradation, which is the case with misfolded proteins in the secretory pathway (13). Therefore, we have introduced the term “ally proteins function” to distinguish between these two roles. In addition to matriptase, the serine proteases transmembrane serine protease 13 (Tmprss13) (17) and hepsin (18), also display a need for an ally protein, of which only HAI-1 and HAI-2 are currently known.

In the present study, we show that a mutation in the start methionine codon of the matriptase gene results in the expression of a truncated, immunoreactive, plasma membrane-bound matriptase variant displaying enzymatic activity.

## Results and discussion

### Matriptase M1I variant is detectable by antibodies against wild-type matriptase

The M1I variation disrupts the start codon used for the initiation of translation of wild-type matriptase. In such cases, the ribosomes continue their scanning of the mRNA sequence downstream of the original start codon in search of an alternative methionine codon that can serve as a new start site for translation (19, 20). To express a matriptase-like protein, translation of the matriptase M1I variant must be initiated from a methionine codon positioned in the same reading frame as the start codon used by wild-type matriptase. There are six methionine codons besides the start codon used by wild-type matriptase that could potentially serve as alternative start codons for matriptase M1I (Fig. 1). The resulting amino-terminally truncated protein may have matriptase immunoreactivity if the epitope in question is located within the open reading frame encoding the matriptase M1I protein product.

To investigate whether an immunoreactive matriptase M1I protein is expressed, HEK293 cells were transiently transfected with expression plasmids encoding matriptase M1I, either alone or co-transfected with either HAI-1 or HAI-2. Two days post-transfection, cell lysates were prepared and analyzed by Western blotting using two different primary antibodies against matriptase (Fig. 1). As controls, wild-type matriptase, zymogen-locked matriptase (R614A), active-site-mutated matriptase (S805A), and another ichthyosis-causing matriptase variant (G827R) (6) were analyzed in the same manner.

Wild-type matriptase is synthesized in a full-length non-SEA domain-cleaved form, which auto-cleaves to generate the SEA domain-cleaved form by cleavage after the glycine amino acid residue in position 149 (Fig. 1). For wild-type matriptase, both the non-SEA domain-cleaved and the SEA domain-cleaved forms could be detected, but with more intense bands when co-expressed with either HAI-1 or HAI-2 (Fig. 2, lanes 1–3). This confirms that wild-type matriptase needs an ally protein, such as either HAI-1 or HAI-2, to stabilize the protease (13, 16), as HEK293 cells only express a low amount of HAI-2 endogenously, which can be detected if overexposed (data not shown). HEK293 cells do not express detectable levels of HAI-1 (Fig. 2).

**Figure 2:**
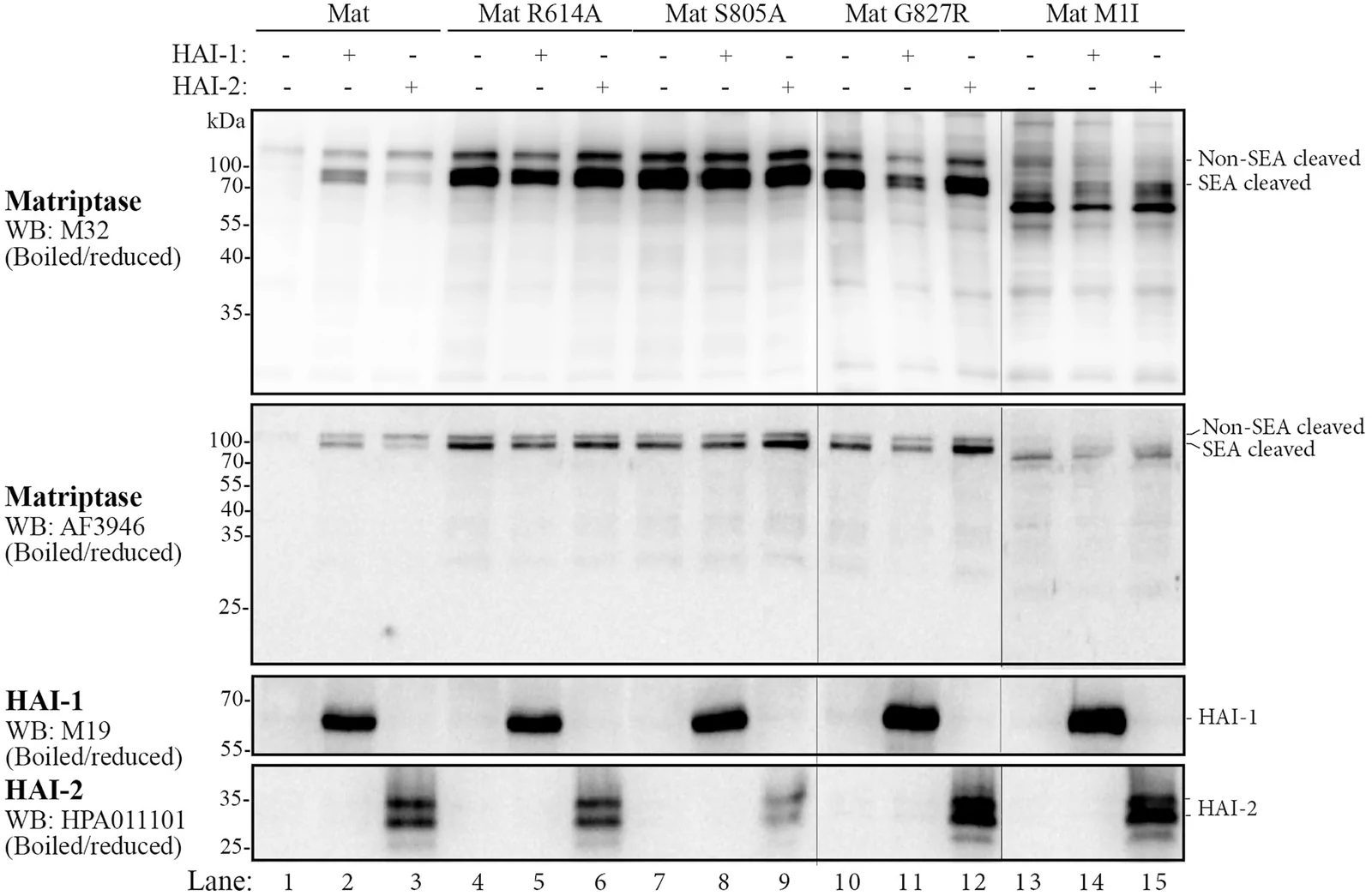
**The matriptase M1I variant is expressed as a truncated variant of matriptase and can be detected by antibodies against the matriptase LDLR and SPD domains.** HEK293 cells were transfected with expression plasmids encoding wild-type matriptase (Mat), zymogen-locked matriptase (Mat R614A), active-site-mutated matriptase (Mat S805A), an ichthyosis-causing matriptase variant (Mat G827R), or the matriptase M1I variant (Mat M1I), either alone or together with HAI-1 or HAI-2, as indicated. After 48 h, the cell lysates were harvested and analyzed by Western blot. Wild-type matriptase can be detected as two bands representing the full-length (non-SEA-cleaved) and SEA domain-cleaved (SEA-cleaved) form of the protein as indicated to the *right*.

Matriptase R614A, S805A, and G827R could all be detected in both the non-SEA domain-cleaved form and the SEA domain-cleaved form, independently of whether they were expressed alone or co-expressed with either HAI-1 or HAI-2 (Fig. 2, lanes 4–12). It has previously been shown that matriptase mutants R614A, S805A, and G827R are expressed independently of the presence of an ally protein such as HAI-1 or HAI-2 (13).

When the lysates of cells transfected with matriptase M1I were analyzed, both antibodies against matriptase, M32 and AF3946, were able to detect immunoreactive bands in both the absence and the presence of co-expressed HAI-1 or HAI-2 (Fig. 2, lanes 13–15). The immunoreactive bands detected in lysates of cells transfected with matriptase M1I migrated further in the gel than bands from the non-SEA domain-cleaved and SEA domain-cleaved matriptase found in lysates from cells expressing wild-type matriptase, matriptase R614A, S805A, or G827R (Fig. 2, lanes 4–12), indicating a lower molecular weight of matriptase M1I compared to the wild-type matriptase.

The matriptase M1I protein product could be detected using two different antibodies targeting matriptase, showing that M1I is expressed using the same open reading frame as wild-type matriptase. Matriptase M1I contains the third LDLR domain and the serine protease domain of matriptase, since it can be detected by antibodies binding to these domains, M32, and AF3946 (Fig. 1).

In conclusion, the M1I variant is expressed as an amino-terminal truncated version of matriptase.

### Matriptase M1I can be detected as a plasma membrane-bound protein

Matriptase knock-out mice die shortly after birth, showing that matriptase is necessary for survival (1). The immunoreactivity of the matriptase M1I protein product shows that this protein variant is indeed expressed. Nonetheless, in order to replace the vital function of wild-type matriptase, matriptase M1I would need to localize to the same subcellular compartments as wild-type matriptase, which are the secretory pathway and the plasma membrane (Fig. 3*A*) (9). The open reading frame of wild-type matriptase contains six methionine codons that could potentially be used as alternative start codons for matriptase M1I, including: M94, M173, M180, M226, M285, and M337 (Fig. 1). Initiation of translation from any of these methionine codons would result in a truncated matriptase variant that is devoid of the cytoplasmic tail and transmembrane anchor (21) formed by amino acid residues 1 to 76 of wild-type matriptase (Fig. 1). This amino terminal truncated matriptase M1I variant would not be expected to undergo co-translational translocation across the rER membrane. Instead, the matriptase M1I protein would be expected to be released into the cytosol from a ribosome not attached to the ER (Fig. 3*B*). Alternatively, translation initiation from another start site could cause the M1I matriptase variant to activate a normally hidden or unused (cryptic) signal peptide or membrane anchor. The activation of a cryptic signal peptide or membrane anchor would enable co-translational translocation of matriptase M1I across the rER membrane, whereby access to the secretory pathway and the plasma membrane would be regained as the lost membrane anchor is replaced by a signal peptide or membrane anchor with an equivalent function. Insertion of matriptase M1I into the membrane by a cryptic signal peptide may occur in both orientations, namely, either the amino terminus on the cytosolic side of the rER membrane (Fig. 3*C*) or the amino terminus on the luminal side of the rER membrane (Fig. 3*D*), as the orientation depends on the distribution of charged amino acid residues around the signal peptide (20, 22). The ability of matriptase M1I to enter the secretory pathway therefore depends on whether the amino acid residues following the start methionine(s) chosen by the ribosome contain a sequence that can function as a signal peptide or a membrane anchor.

**Figure 3:**
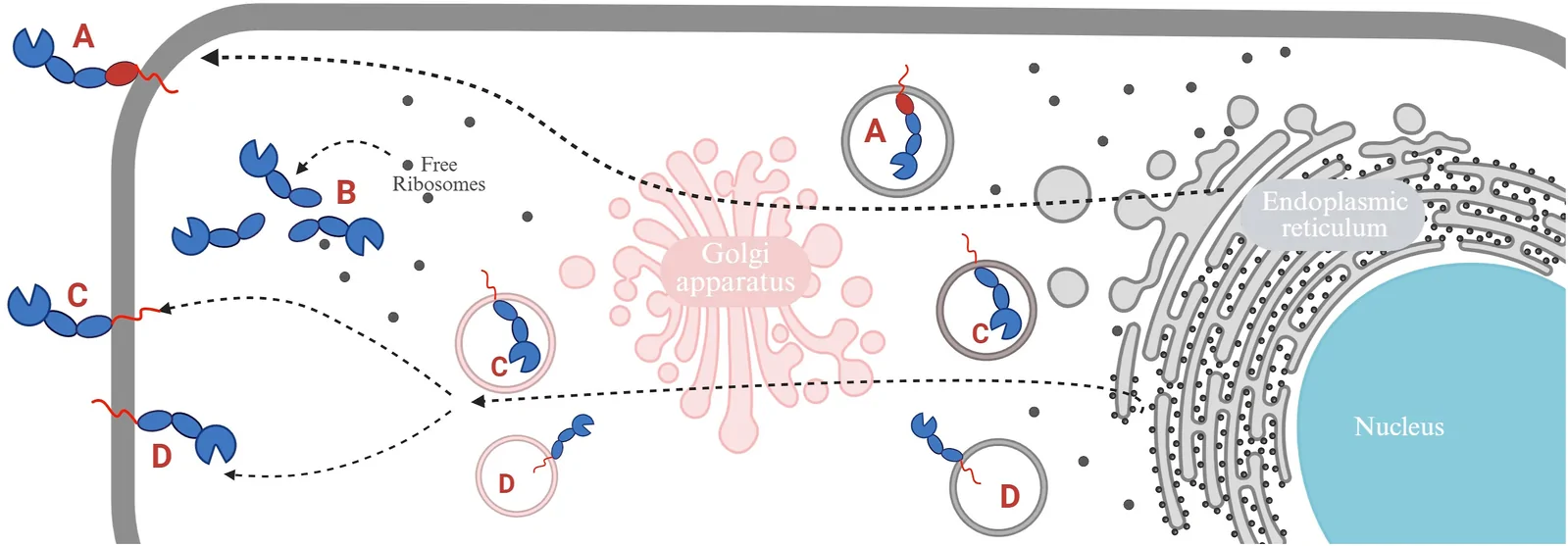
**Subcellular localization and intracellular transport of wild-type matriptase and three possible scenarios for the intracellular targeting and transport of the matriptase M1I protein product.***A*, wild-type matriptase is synthesized with an amino-terminal non-cleavable membrane anchor ensuring its co-translational insertion into the rER. Wild-type matriptase is subsequently transported through the secretory pathway to the plasma membrane. *B*, the amino-terminally truncated matriptase M1I translated from an alternative start codon not succeeded by an amino acid sequence that resembles a signal peptide (a cryptic signal peptide) or a membrane anchor will be synthesized on free ribosomes and then released into the cytosol. *C* and *D*, illustrate the two possible orientations of matriptase M1I containing a cryptic signal sequence downstream of the alternative start codon. The orientation of the protein on the rER membrane, and ultimately on the plasma membrane, will depend on the distribution of the charged amino acid residues located in the protein surrounding the signal peptide.

To investigate whether matriptase M1I can be detected on the plasma membrane, cell surface biotinylation was used to label all plasma membrane-bound proteins using a non-membrane-permeant biotinylation reagent. Biotinylated proteins were isolated by streptavidin pulldown and analyzed by Western blotting.

We have previously shown that the matriptase mutation G149E prevents SEA-domain cleavage of matriptase, resulting in a protein product that is transported to the plasma membrane only when co-expressed with HAI-1 and/or HAI-2 (16). We have also shown that matriptase G149E co-expressed with HAI-1 and HAI-2 is found only in low abundance on the plasma membrane (16). Matriptase G149E co-expressed with HAI-1 and HAI-2 contains amino acid residues 1 to 855 of wild-type matriptase, in other words, all 855 amino acid residues of wild-type matriptase.

We have also previously shown that active-site-mutated matriptase S805A is an abundant protein that undergoes SEA-domain cleavage, but only the SEA-domain-cleaved form is transported to the plasma membrane (16). Plasma membrane-bound matriptase S805A thus contains amino acid residues 150 to 855 of wild-type matriptase due to SEA-domain cleavage, corresponding to 706 amino acid residues.

HEK293 cells were transiently transfected with an expression plasmid encoding matriptase G149E together with HAI-1 and HAI-2, matriptase M1I, or active-site-mutated matriptase S805A. The HEK293 cells were biotinylated 48 h post-transfection using NHS-SS-Biotin, followed by lysis of the cells and streptavidin pulldown. The precipitates were then analyzed by Western blotting; however, for matriptase S805A, only one-quarter of the precipitate was analyzed when compared to the other samples (Fig. 4).

**Figure 4:**
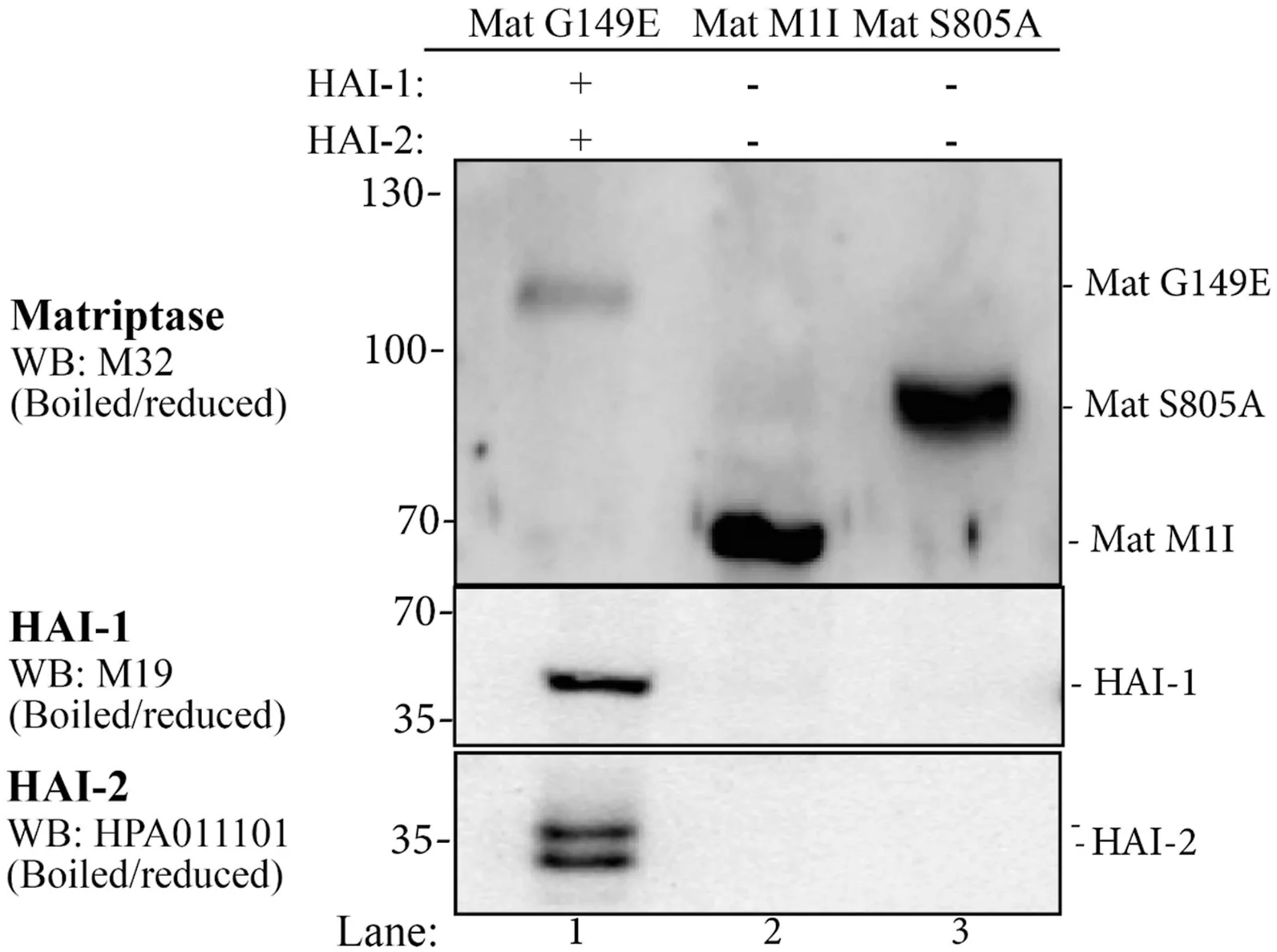
**Matriptase M1I can be detected on the plasma membrane.** HEK293 cells were transfected with matriptase G149E co-expressed with HAI-1 and HAI-2, matriptase M1I or matriptase S805A as indicated. Forty-eight hours after transfection, cells were biotinylated using a non-cell-permeant biotinylation reagent, lysed, and precipitated with streptavidin. The precipitates were analyzed by Western blotting. All samples were boiled and reduced. Primary antibodies directed against matriptase, HAI-1 and HAI-2 were used as indicated on the *left*. The figure shows a representative experiment out of three.

Matriptase M1I could be detected as an immunoreactive band after cell surface biotinylation and streptavidin pulldown. The fact that matriptase M1I can be biotinylated and pulled down using streptavidin shows that matriptase M1I is attached to the plasma membrane. The M1I displayed a molecular weight of 67 kD, which corresponds to 609 amino acid residues assuming an average molecular weight of 110 D for amino acid residues. The number of amino acids in M1I is bound to vary depending on which start methionine are used thus M94, M173, M180, M226, M337 would generate a protein of 762, 683, 676, 630, 571 and 519 amino acid residues, respectively. As matriptase is N-glycosylated it is not likely to migrate according to its protein content and thus we can only conclude that M1I is smaller than Matriptase S805A.

Matriptase G149E co-expressed with HAI-2 and HAI-2 and matriptase S805A displayed immunoreactive bands with slower mobility than matriptase M1I (Fig. 4), showing that matriptase MI1 contains less than 706 amino acid residues. As only a quarter of matriptase S805A precipitate was analyzed compared to the other samples, matriptase M1I is thus more abundant than matriptase G149E and less abundant than matriptase S805A on the plasma membrane. Matriptase S805A displayed a molecular weight of 90 kDa, and Matriptase G149E displayed a molecular weight of 107 kDa. Both Matriptase S805A and Matriptase G149E migrate with a higher molecular weight than expected based on their primary structure, probably due to N-glycosylation at several positions. However, their primary structure differs by 150 amino acid residues, which corresponds well with the difference in their apparent molecular weight of 107 kDa−90 kDa = 17 kDa, corresponding to 154 amino acid residues.

In conclusion, these results strongly suggest that the matriptase M1I variant is a membrane bound protein with transport competence to reach the plasma membrane *via* the secretory pathway.

### Bioinformatics predicts the presence of a cryptic signal peptide in matriptase

The observation that the matriptase M1I protein can be detected on the plasma membrane suggests that an otherwise unused (cryptic) signal peptide is activated by the shift to another start methionine. Signal peptides display a consensus structure consisting of a methionine amino acid residue that serves as the start codon, followed by three conserved regions known as the N-, H-, and C-regions, each characterized by specific features (23) (Fig. 5).

**Figure 5:**
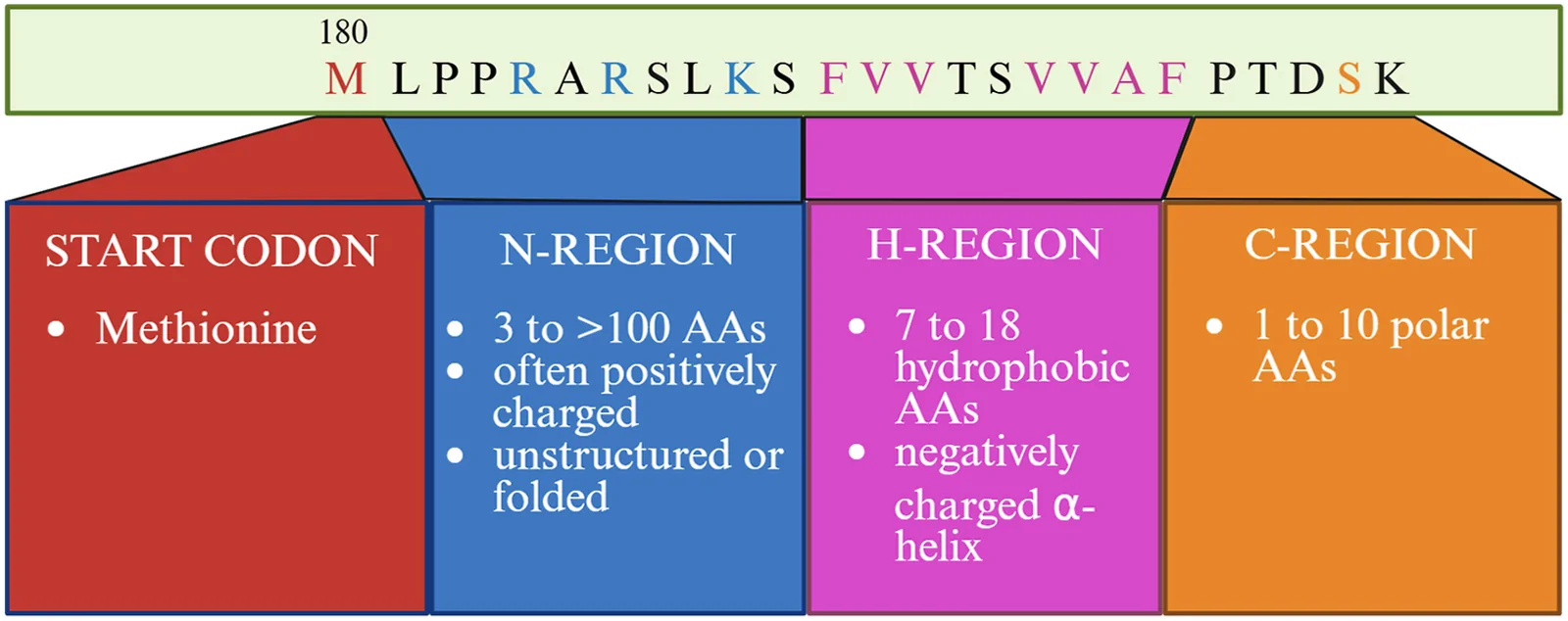
**The Phobius software predicts a non-cleavable signal peptide downstream of M180.** The amino acid sequence downstream of M180 (wild-type matriptase numbering) is colored according to the regions of a standard signal peptide sequence, which includes a start methionine and N-, H-, and C-region, in *blue*, *magenta*, and *yellow*, respectively.

The Phobius software (https://phobius.sbc.su.se/) (24) was used to predict the localization, topology, and cleavability of putative signal peptides following the six methionine amino acid residues located in the open reading frame of wild-type matriptase downstream of the start codon. As a control, the sequence following the start codons of wild-type matriptase, HAI-1, and HAI-2 were analyzed in the same manner. Membrane-bound wild-type matriptase has a membrane anchor instead of a signal peptide (21), whereas HAI-1 and HAI-2 are reported to have a cleavable signal peptide and a carboxy-terminal membrane anchor (25, 26).

Using the Phobius software, a non-cleavable signal peptide was predicted to follow the methionine in position 180 of wild-type matriptase (Table 1) with a structure like a classical signal peptide, as depicted (Fig. 5), and with the same topology as wild-type matriptase. Cleavable signal peptides were predicted to be located following M1 in HAI-1 and HAI-2, whereas no signal peptides could be detected following M1, M94, M173, M226, M285, or M337 in matriptase. This suggests that matriptase M1I uses M180 as the start codon to produce a membrane-bound, amino-terminal truncated matriptase M1I variant. Matriptase M1I is predicted to span from amino acid residue 180 to 855 of wild-type matriptase, a total of 676 amino acid residues. The migration of matriptase M1I on Western blot (Fig. 4) is only very roughly compatible with a protein of 676 amino acid residues. However, M1I is probably N-glycosylated, as is wild-type matriptase, which may make up for the discrepancy. Matriptase M1I is predicted to have the same topology as wild-type matriptase, which makes it possible that HAI-1 and HAI-2 can inhibit the enzymatic activity of the matriptase M1I variant and act as ally proteins towards matriptase M1I, as seen for wild-type matriptase. In conclusion, a possible cryptic signal sequence in matriptase M1I located shortly after M180 was identified using bioinformatics.

**Table 1:** A Signal Peptide Following Methionine in Position 180 Can Be Predicted Using the Phobius Software

Protein	Codon	Prediction of signal Peptide?	Prediction of cleavage site in signal peptide?
Matriptase	M1	No^a^	NA
Matriptase	M94	No	NA
Matriptase	M173	No	NA
Matriptase	M180	Yes	No
Matriptase	M226	No	NA
Matriptase	M285	No	NA
Matriptase	M337	No	NA
HAI-1	M1	Yes	Yes
HAI-2	M1	Yes	Yes

a Wild-type matriptase contains a signal anchor sequence (22), instead of a signal peptide.

### Matriptase M1I displays peptidolytic activity

Presumably, the matriptase M1I variant requires correct folding to be able to fulfill the essential biological role of wild-type matriptase. Displaying proteolytic activity is a strong indication that a protein has obtained its correct fold. In the present study, the matriptase-specific inhibitory antibody aZ-mAb-6, previously described by us (27), was used to distinguish between general serine protease activity and matriptase-specific peptidolytic activity. We have previously shown that the HAI-2 mutant R48L lacks the ability to inhibit matriptase (12), yet is still able to provide the necessary ally protein function to stabilize matriptase protein expression in cells (16).

To assess the enzymatic activity of the matriptase M1I variant, HEK293 cells were transiently transfected with expression plasmids encoding HAI-2 R48L alone or together with either the wild-type or mutants/variants of matriptase; R614A, S805A, G827R, or M1I. Forty-eight hours post transfection, the HEK293 cells were lysed, and the cell lysates were incubated with or without the aZ-mAb-6 antibody, which specifically inhibits both zymogen and activated matriptase (27). Mock-transfected cells were assessed in the same way and included as a control. The peptidolytic activity of the cell lysates was then determined as the rate of cleavage of the unspecific serine protease peptide substrate (S-2288). The matriptase-specific activity was determined as the difference between the rate of cleavage in the presence and absence of the aZ-mAb-6 antibody (Fig. 6).

**Figure 6:**
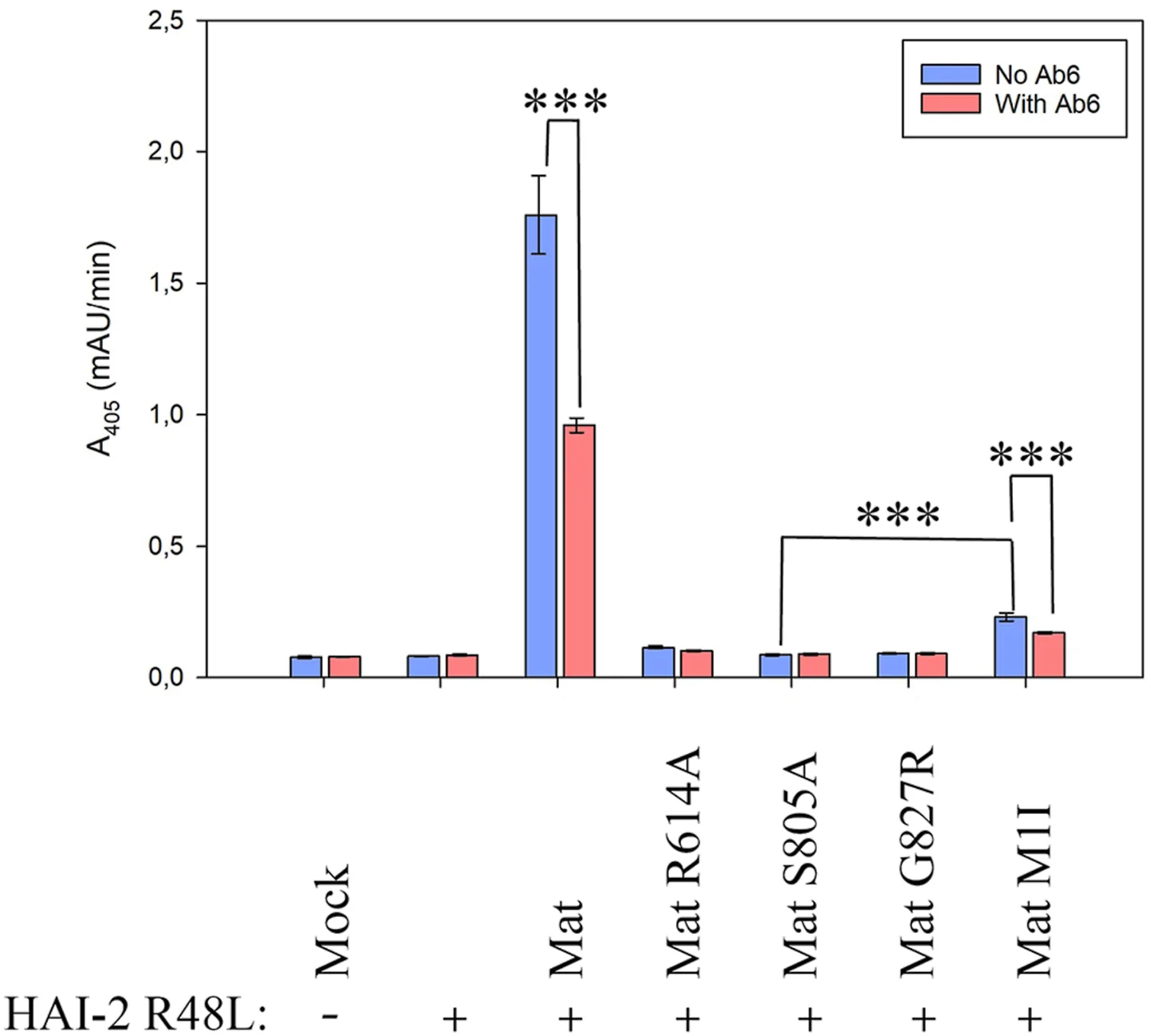
**Matriptase M1I displays matriptase-specific peptidolytic activity.** HEK293 cells were transiently transfected with various matriptase variants in combination with HAI-2 R48L or HAI-2 R48L alone, as indicated. HAI-2 R48L is an inefficient inhibitor of matriptase but is able to provide ally protein function to stabilize the matriptase protein. Cell lysates were incubated with or without the matriptase inhibitory antibody, aZ-mAb-6, and subsequently the unspecific serine protease substrate S2288 was added. Peptidolytic activity was calculated as the rate of absorbance change at 405 nm resulting from hydrolysis of the chromogenic substrate, SS2288. Matriptase-specific peptidolytic activity was estimated as the difference in peptidolytic activity in the absence *versus* presence of aZ-mAb-6 antibody. Each column represents the mean ± SD peptidolytic activity (mAU/min) in the absence (*blue columns*) or presence (*red columns*) of aZ-mAb-6 antibodies (Ab6). Significant differences in the turnover rate are indicated with the corresponding *p*-values (∗∗∗; *p* ≤ 0.001 by Student's *t* test. N ≥ 3).

A low level of peptidolytic activity could be detected in the lysates of cells transfected with either a mock plasmid or the HAI-2 R48L expression plasmid alone. No matriptase activity could be detected in these lysates, as the peptidolytic activity was unaffected by the presence of aZ-mAb-6 antibody (Fig. 6). These control samples indicate the background level of peptidolytic activity derived from endogenously expressed proteases. The lysate from cells co-expressing HAI-2 R48L and wild-type matriptase displayed a high level of peptidolytic activity, which significantly decreased upon treatment with the aZ-mAb-6 antibody (Fig. 6). This confirms that the HAI-2 R48L mutant is an inefficient inhibitor of matriptase, but able to function as an ally protein. When HAI-2 R48L was co-expressed with either zymogen-locked matriptase (R614A), active-site-mutated matriptase (S805A), or the ichthyosis-causing matriptase variant (G827R), background levels of peptidolytic activity were detected regardless of whether the inhibitory antibody aZ-mAb-6 was absent or present. This shows that these matriptase variants/mutants exhibit no or undetectable levels of matriptase peptidolytic activity. The lysate from cells co-expressing HAI-2 R48L and matriptase S805A serves as the best indicator of the background level of peptidolytic activity, as this is a proteolytically dead matriptase mutant. The cell lysate from cells co-expressing HAI-2 R48L and matriptase M1I exhibited a peptidolytic activity significantly above that of the lysate from cells co-expressing HAI-2 R48L and the active-site-mutated matriptase S805A (*p* ≤ 0.001) (Fig. 6). Furthermore, the proteolytic activity of the cell lysate from cells co-expressing matriptase M1I and HAI-2 R48L significantly decreased upon treatment with the aZ-mAb-6 antibody, showing that the matriptase M1I variant protein displays matriptase-specific peptidolytic activity (Fig. 6). In conclusion, the matriptase M1I protein displays enzymatic activity, showing that at least the SPD domain obtained the correct fold.

### The matriptase M1I variant is dependent on ally proteins for cell surface expression

To examine whether matriptase M1I, like wild-type matriptase, requires an ally protein for expression on the plasma membrane, immunohistochemistry was used to analyze recombinant expression in HEK293, known to have a low endogenous expression of HAI-2 detectable by Western blot only if overexposed (data not shown).

HEK293 cells were transiently transfected with expression plasmids encoding matriptase M1I or matriptase S805A in the absence or presence of HAI-1, HAI-2, or both HAI-1 and HAI-2. The cells were fixed and stained using immunohistochemistry in the presence or absence of Triton X-100 to distinguish between matriptase on the plasma membrane (in the absence of Triton-X-100) and total matriptase (in the presence of Triton X-100) (Fig. 7).

**Figure 7:**
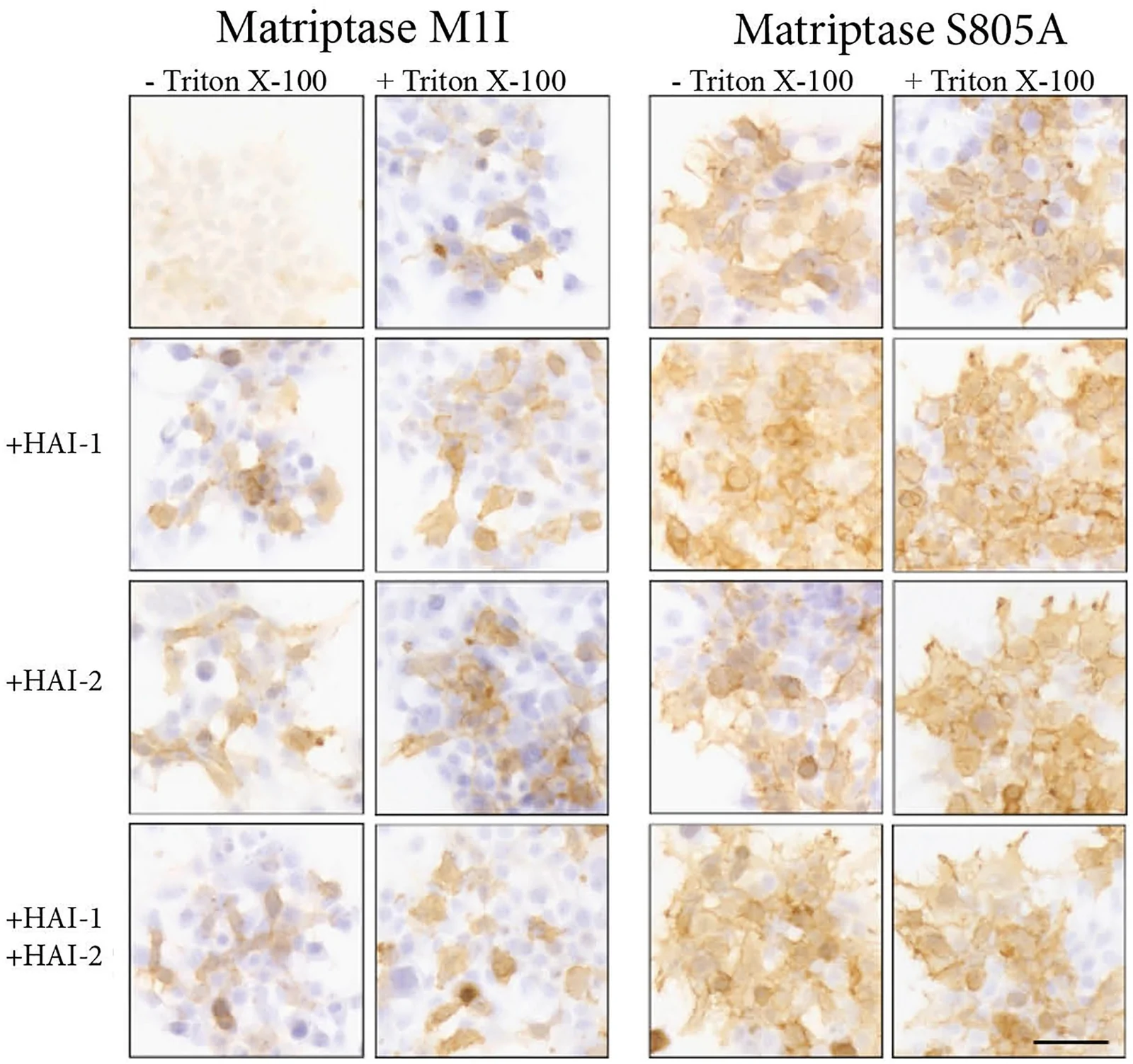
**The M1I matriptase variant uses an ally protein for localization on the plasma membrane.** HEK293 cells were transiently transfected with the matriptase M1I variant or matriptase S805A alone or together with HAI-1 and/or HAI-2 as indicated. Cells were fixed and stained for matriptase either in the absence or presence of Triton X-100. Bar 50 μm. The figure shows a representative experiment out of three.

In cells expressing matriptase S805A, matriptase staining could be detected in all cells regardless of whether matriptase was expressed alone or with HAI-1 and/or HAI-2, and regardless of whether Triton X-100 was present or absent. This shows that matriptase S805A is expressed on the plasma membrane independently of the presence or absence of HAI-1 and/or HAI-2.

In the absence of Triton X-100, there was a clearly increased detection of cell surface matriptase M1I when co-expressed with HAI-1 and/or HAI-2 compared with matriptase M1I expressed alone. In contrast there was no detectable difference between matriptase M1I expressed in the absence or presence of HAIs in the presence of Triton X-100. This suggests that matriptase M1I is dependent on an ally protein like HAI-1 or HAI-2 for cell surface expression of the matriptase M1I variant protein.

### Matriptase M1I is likely to replace the essential functions of wild-type matriptase

The present study focuses on whether matriptase has an essential function in humans, as seen in mice. Matriptase knock-out mice die shortly after birth. The cause of death is unclear, but dehydration due to a permeable skin barrier has been suggested, which points to a defect in the keratinocytes. The mechanism targeting proteins to the ER is a well-researched topic, and the mechanisms are not reported to differ between cell types. This study used the fibroblast cell line, HEK293, as model cells, even though matriptase is expressed in most epithelial cells but not in fibroblasts (28). Thus, all conclusions drawn from this study is based on the assumption that the ER-targeting mechanisms do not differ between cell types and especially not between keratinocytes and fibroblasts.

Based on our findings we propose that matriptase M1I is expressed, most likely using the methionine codon in position 180 of the wild-type matriptase as an alternative start codon, and thus employs a cryptic non-cleavable signal peptide to ensure co-translational translocation into the rER similar to the scenario depicted in (Fig. 3*C*). In support of this, the protein product of matriptase M1I can be detected using two different antibodies against wild-type matriptase. Furthermore, the fact that matriptase M1I displays matriptase specific enzymatic activity, as well as being transport competent down the secretory pathway and detectable on the plasma membrane, strongly suggests that it is able to obtain the correct fold. Based on this, we suggest that matriptase M1I is expressed as a functional protein with correct subcellular localization and likely fully capable of replacing the essential function of wild-type matriptase. At the same time some aspects of Matriptase M1I's function seem deficient in an unknown manner as it causes ARCI11. An elucidation of the molecular mechanisms behind ARCI11 is beyond the scope of the present study.

If the methionine in position 180 is used as a start codon, then the domain arrangement in the matriptase M1I protein differs from wild-type matriptase in several ways. Amino acid residues 180 to 203 (matriptase wild-type numbering) form part of the SEA domain in wild-type matriptase, but in matriptase M1I they form the N-terminal cytoplasmic tail and the transmembrane domain. Matriptase M1I thus contains a cytoplasmic tail and a transmembrane domain, two CUB domains, four LDLR domains, and a C-terminal SPD.

Matriptase is essential and thus likely contains one or more essential domain(s); but which one? In the present paper it can be deduced that in humans, matriptase devoid of the cytoplasmic tail, the transmembrane domain, and the SEA domain results in ichthyosis which rules out these domains as essential. Another person with ichthyosis has been reported to carry a frameshift mutation in the SPD domain, causing deletion of most of this domain (3), which rules out the serine protease domain as essential. Likewise, another person with ichthyosis had a stop codon in position 415 (29). Theoretically, this mutation rules out the LDLR domains as essential. This suggests that the essential feature is to be found among the two CUB domains.

This M1I mutation is a 'start loss' pathogenic variant. Many “pathogenic” start-loss variants have been described in the literature; some of these disturb amino-terminal signals guiding intracellular transport. However, we have not found other examples where the methionine selected by the ribosome for initiation of translation “activates” a signal peptide or other transport signal not normally in use.

## Experimental procedures

### Cell culturing, transfections and DNA constructs

The human embryonic kidney cell line HEK293 was cultured in Dulbecco’s Modified Eagle’s Medium 1965 supplemented with 1% L-glutamine, 10% fetal bovine serum and 1% penicillin/streptomycin at 37 °C in an atmosphere of 5% CO_2_. For experiments, cells grew to 80% confluence. One day prior to transfection, 500.000 to 600.000 cells per well were seeded into 6-well Nunclon Cell-Culture Treated Multidishes (cat. no. 140675). HEK293 cells were transiently transfected using either Lipofectamine 2000 (Invitrogen), Lipofectamine 3000TM (Invitrogen) or Metafectene (Biontex) in accordance with the recommendations from the manufacturer. In the case of co-transfections, the same amount of each individual plasmid was applied, and empty plasmids were added to ensure that the same total amount of plasmid was used for each transfection. Full-length human matriptase, HAI-1 and HAI-2 cDNAs incorporated into pCDNA3.1 plasmid vectors were used. The HAI-2 cDNA includes a naturally occurring SNP, rs11548457, which generates a V200L amino acid substitution. The M1I mutation was introduced into the matriptase-encoding cDNA by site-directed mutagenesis using the GeneArt Site-Directed Mutagenesis System (cat.no. A13282, Life Technologies) The mutated cDNAs obtained were verified by sequencing. The remaining expression plasmids used in this study have been previously described: matriptase HAI-1, HAI-2, matriptase, matriptase S805A, matriptase R614A, matriptase G827R, matriptase G149E (16). Forty-eight hours post-transfection, cells were lysed in lysis buffer (PBS, 1% Triton X-100 and 0.5% SDS). The cell extracts were then centrifuged for 40 min at 20.000 x g and the supernatants were stored at −20 °C.

### SDS-PAGE and Western blotting

Samples were prepared by the addition of4 × NuPAGE LDS Sample Buffer (Invitrogen) and for reducing conditions 10 × NuPAGE Sample Reducing Agent. The samples were subsequently boiled at 100 °C for 5 min. Proteins were separated on NuPAGE TM 10% Bis-Tris Midi Gels (cat.no. WG1201B0X, Invitrogen) and transferred to PVDF Transfer Membranes (Thermo Scientific). The membranes were then blocked with PBS containing 0.1% Tween-20 and 10% skimmed milk powder (10% MPBS-T) for 0.5 to 1 h at room temperature. Each membrane was subsequently incubated with primary antibody diluted in 1% MPBS-T at 4 °C overnight. The membranes were then washed 3 × 5 min in PBS-T and incubated with horseradish peroxidase (HRP)-conjugated secondary antibodies diluted in 1% MPBS-T for 1 h at room temperature. Following another 3 × 5 min wash in PBS-T, the membranes were developed using the WesternBrightTM Sirius Western blotting detection kit (Advansta) according to the protocol supplied by the manufacturer. The membranes were visualized using a Fuji LAS-1000 camera and Intelligent DarkBox II (FujiFilm Sweden AB) and the program Image Reader LAS-1000 Lite V1.5.

### Antibodies

Following transfer, the membranes were incubated with one of the primary antibodies: polyclonal sheep anti-human matriptase IgG (cat.no. AF3946, R&D systems) (1:1000) or polyclonal rabbit anti-human HAI-2 antibody (cat.no. HPA011101, Sigma Prestige Antibodies) (1:1000). For the peptidolytic activity assay, the monoclonal aZ-mAb-6 antibody (39) was used to inhibit the proteolytic activity of matriptase.

### Cell surface protein biotinylation

Transiently transfected HEK293 cells in 6-well culture plates were washed twice with ice-cold PBS+ (PBS supplemented with 0.7 mM CaCl_2_ and 0.25 mM MgCl_2_). For labelling of surface proteins, cells were surface biotinylated with 1 mg/ml EZ-link Sulfo-NHS-SS-Biotin (prod. no. 21331, Thermo Scientific) in PBS+ for 30 min at 4 °C. Following a wash in PBS+, residual biotin was quenched for 5 min at 4 °C with 50 mM glycine in PBS and the cells were washed with PBS+ before lysis as described above. Biotinylated cell surface proteins were precipitated from the cell extracts by addition of 75 ml streptavidin-coated resin (prod. no. 20349 Thermo Scientific), prepared as described by the manufacturer, and incubated overnight with end-over-end rotation at 4 °C. The resin was pulled down (2000×*g*, 30 s) and washed four times with 25 mM Tris–HCl, 500 mM NaCl, 0.5% Triton X-100 pH 7.8 and three times with 10 mM Tris–HCl, 150 mM NaCl, pH 7.8. Precipitated proteins were eluted by boiling for 10 min in SDS sample buffer (2 mM Tris–HCl pH 6.8, 0.2% SDS, 2% glycerol, 0.2 mM EDTA, 0.01% bromophenol blue) and subsequently analysed by SDS–PAGE and western blotting alongside the cell extracts.

### Immunocytochemistry

Twenty-four hours before transfection, HEK293 cells were seeded onto polylysine-coated glass coverslips in 6-well Nunclon Cell-Culture Treated Multidishes (cat. no. 140675). For IHC, cells were transiently transfected using Lipofectamine 3000 (Invitrogen). After 48 h, the HEK293 cells were fixed in 4% formaldehyde prepared in PBS (Invitrogen) for 20 min at room temperature before being washed twice with PBS. The coverslips were then washed 3 × 5 min in wash buffer composed of Tris-buffered saline (TBS) (150 mM NaCl, 50 mM Tris-HCl, pH 7.6) with 0.01% Triton X-100. Subsequently, the coverslips were incubated in 10% goat serum for 30 min followed by 1 h incubation in primary antibody (M32) diluted 1:2000 in goat serum. The coverslips were then washed 3 × 5 min in wash buffer and incubated with HRP-conjugated secondary antibody (Dako EnVision+ System-HRP Labelled Polymer Anti-Rabbit) in goat serum for 30 min. Following another 3 × 5 min wash in wash buffer, the coverslips were incubated with 3,3′- diaminobenzidine (DAB) (EnVision) for 10 min. Subsequently, the coverslips were washed 2 × 5 min with wash buffer and 1 × 5 min with Millipore water before the cell nuclei were stained with hematoxylin (AMPQ00253, Ampliqon) for 30 s. The coverslips were then washed in Millipore water for 5 min before being mounted onto glass slides using Aquamount (cat. no. 36086, BDH). Images were acquired on a Leica DM5500 microscope equipped with a Leica DMC6200 camera.

### Peptidolytic activity assay

Cell extracts of transiently transfected HEK293 cells were subjected to a peptidolytic activity assay. For this assay, H-d-Isoleucyl-l-prolyl-l-arginine-p-nitroaniline (cat.no. S-2288, Chromogenix) was used as a chromogenic substrate, which is cleaved by a broad spectrum of serine proteases. In order to determine the proteolytic activity of matriptase, each sample was investigated in the presence or absence of aZ-mAb-6 antibodies, which specifically inhibit matriptase activity (27). Cell extracts were treated with a similar volume of either PBS or aZ-mAb-6 antibody to a final concentration of 200 nM (corresponding to 10 × Ki of the aZ-mAb-6 antibody (27). The extracts were then incubated at 37 °C for 1 h. Afterward, the cell extracts were treated with 300 μM chromogenic substrate, S-2288, in an equal volume of HBS buffer composed of 20 mM HEPES (pH 7.4), 140 mM NaCl supplemented with 0.1% BSA (Sigma). The resulting color development was measured at 405 nm every min for 5 h on a standard plate reader (Biotek Synergy HT) at 37 °C. The peptidolytic activity of each sample was calculated as the rate of color development (mAU/min). Matriptase-specific activity was determined as the difference between proteolytic activity in the presence and absence of aZ-mAb-6 antibody. The aZ-mAb-6 antibody was subjected to degradation by proteases in the cell extracts. Thus, the peptidolytic assay underestimates the amount of matriptase-specific activity present in the investigated samples.

## Data availability

All data are contained within the manuscript.

## Conflict of interest

The authors declare that they have no conflicts of interest with the contents of this article.

## References

[1] List K., Haudenschild C.C., Szabo R., Chen W., Wahl S.M., Swaim W. (2002). Matriptase/MT-SP1 is required for postnatal survival, epidermal barrier function, hair follicle development, and thymic homeostasis. Oncogene.

[2] Avrahami L., Maas S., Pasmanik-Chor M., Rainshtein L., Magal N., Smitt J. (2008). Autosomal recessive ichthyosis with hypotrichosis syndrome: further delineation of the phenotype. Clin. Genet..

[3] Alef T., Torres S., Hausser I., Metze D., Türsen U., Lestringant G.G. (2009). Ichthyosis, follicular atrophoderma, and hypotrichosis caused by mutations in ST14 is associated with impaired profilaggrin processing. J. Invest. Dermatol..

[4] Ahmad F., Ahmed I., Nasir A., Umair M., Shahzad S., Muhammad D. (2018). A disease-causing novel missense mutation in the ST14 gene underlies autosomal recessive ichthyosis with hypotrichosis syndrome in a consanguineous family. Eur. J. Dermatol..

[5] Youssefian L., Touati A., Saeidian A.H., Zargari O., Zeinali S., Vahidnezhad H. (2017). A novel mutation in ST14 at a functionally significant amino acid residue expands the spectrum of ichthyosis-hypotrichosis syndrome. Orphanet. J. Rare Dis..

[6] Basel-Vanagaite L., Attia R., Ishida-Yamamoto A., Rainshtein L., Ben Amitai D., Lurie R. (2007). Autosomal recessive ichthyosis with hypotrichosis caused by a mutation in ST14, encoding type II transmembrane serine protease matriptase. Am. J. Hum. Genet..

[7] List K., Szabo R., Molinolo A., Sriuranpong V., Redeye V., Murdock T. (2005). Deregulated matriptase causes ras-independent multistage carcinogenesis and promotes ras-mediated malignant transformation. Genes Dev..

[8] Sales K.U., Friis S., Abusleme L., Moutsopoulos N.M., Bugge T.H. (2015). Matriptase promotes inflammatory cell accumulation and progression of established epidermal tumors. Oncogene.

[9] Friis S., Godiksen S., Bornholdt J., Selzer-Plon J., Rasmussen H.B., Bugge T.H. (2011). Transport via the transcytotic pathway makes prostasin available as a substrate for matriptase. J. Biol. Chem..

[10] Sales K.U., Friis S., Konkel J.E., Godiksen S., Hatakeyama M., Hansen K.K. (2015). Non-hematopoietic PAR-2 is essential for matriptase-driven pre-malignant progression and potentiation of ras-mediated squamous cell carcinogenesis. Oncogene.

[11] Szabo R., Rasmussen A.L., Moyer A.B., Kosa P., Schafer J.M., Molinolo A.A. (2011). c-Met-induced epithelial carcinogenesis is initiated by the serine protease matriptase. Oncogene.

[12] Skovbjerg S., Holt-Danborg L., Nonboe A.W., Hong Z., Frost Á.K., Schar C.R. (2020). Inhibition of an active zymogen protease: the zymogen form of matriptase is regulated by HAI-1 and HAI-2. Biochem. J..

[13] Nonboe A.W., Bald Z.H., Vogel L.K. (2022). Understanding HAIs: Ally proteins in the fight against cancer. FEBS J..

[14] Oberst M.D., Chen L.Y., Kiyomiya K., Williams C.A., Lee M.S., Johnson M.D. (2005). HAI-1 regulates activation and expression of matriptase, a membrane-bound serine protease. Am. J. Physiol. Cell Physiol..

[15] Larsen B.R., Steffensen S.D., Nielsen N.V., Friis S., Godiksen S., Bornholdt J. (2013). Hepatocyte growth factor activator inhibitor-2 prevents shedding of matriptase. Exp. Cell Res..

[16] Nonboe A.W., Krigslund O., Soendergaard C., Skovbjerg S., Friis S., Andersen M.N. (2017). HAI-2 stabilizes, inhibits and regulates SEA-cleavage-dependent secretory transport of matriptase. Traffic.

[17] Murray A.S., Varela F.A., Hyland T.E., Schoenbeck A.J., White J.M., Tanabe L.M. (2017). Phosphorylation of the type II transmembrane serine protease, TMPRSS13, in hepatocyte growth factor activator inhibitor-1 and -2-mediated cell-surface localization. J. Biol. Chem..

[18] Jin Z., Zhang Y., Chen W., Li H., Shi L., Wang D. (2025). Intracellular autoactivation and surface location of hepsin, TMPRSS2, and TMPRSS13. Life Sci..

[19] Kozak M. (1989). The scanning model for translation: an update. J. Cell Biol..

[20] von Heijne G., Gavel Y. (1988). Topogenic signals in integral membrane proteins. Eur. J. Biochem..

[21] Takeuchi T., Shuman M.A., Craik C.S. (1999). Reverse biochemistry: use of macromolecular protease inhibitors to dissect complex biological processes and identify a membrane-type serine protease in epithelial cancer and normal tissue. Proc. Natl. Acad. Sci. U. S. A..

[22] Goder V., Spiess M. (2003). Molecular mechanism of signal sequence orientation in the endoplasmic reticulum. Embo j.

[23] von Heijne G. (1986). A new method for predicting signal sequence cleavage sites. Nucleic Acids Res..

[24] Käll L., Krogh A., Sonnhammer E.L. (2005). An HMM posterior decoder for sequence feature prediction that includes homology information. Bioinformatics.

[25] Shimomura T., Denda K., Kitamura A., Kawaguchi T., Kito M., Kondo J. (1997). Hepatocyte growth factor activator inhibitor, a novel Kunitz-type serine protease inhibitor. J. Biol. Chem..

[26] Kawaguchi T., Qin L., Shimomura T., Kondo J., Matsumoto K., Denda K. (1997). Purification and cloning of hepatocyte growth factor activator inhibitor type 2, a Kunitz-type serine protease inhibitor. J. Biol. Chem..

[27] Tamberg T., Hong Z., De Schepper D., Skovbjerg S., Dupont D.M., Vitved L. (2019). Blocking the proteolytic activity of zymogen matriptase with antibody-based inhibitors. J. Biol. Chem..

[28] List K., Szabo R., Molinolo A., Nielsen B.S., Bugge T.H. (2006). Delineation of matriptase protein expression by enzymatic gene trapping suggests diverging roles in barrier function, hair formation, and squamous cell carcinogenesis. Am. J. Pathol..

[29] Taghavi-Basmenj M., Razipour M., Davoudi-Dehaghani E., Nasimi M., Abghari F.Z., Karimipoor M. (2019). Identification of a novel mutation in the ST14 gene in an Iranian family with ichthyosis and hypotrichosis. Dermatol. Online J..

